# Dissemination of IncQ1 Plasmids Harboring NTE_KPC_-IId in a Brazilian Hospital

**DOI:** 10.3390/microorganisms13010180

**Published:** 2025-01-16

**Authors:** Camila Maria dos Santos Boralli, Julian Andres Paganini, Rodrigo Silva Meneses, Camila Pacheco Silveira Martins da Mata, Edna Marilea Meireles Leite, Anita C. Schürch, Fernanda L. Paganelli, Rob J. L. Willems, Ilana L. B. C. Camargo

**Affiliations:** 1 Laboratório de Epidemiologia e Microbiologia Moleculares—LEMiMo, Instituto de Física de São Carlos, Universidade de São Paulo, São Carlos 13563-120, SP, Brazil; camila.boralli@gmail.com; 2 University Medical Center Utrecht, 3584 CX Utrecht, The Netherlands; j.a.paganini@uu.nl (J.A.P.); r.silvameneses@umcutrecht.nl (R.S.M.); a.c.schurch@umcutrecht.nl (A.C.S.); fernandapaganelli@hotmail.com (F.L.P.); rwillems@umcutrecht.nl (R.J.L.W.); 3 Risoleta Tolentino Neves Hospital, Belo Horizonte 31744-012, MG, Brazil; camila.mata@hrtn.fundep.ufmg.br (C.P.S.M.d.M.); edna.leite@hrtn.fundep.ufmg.br (E.M.M.L.)

**Keywords:** *bla*_KPC_, NTE_KPC_-IId, plasmid, IncQ1, conjugation rate, plasmid copy number

## Abstract

KPC is a clinically significant serine carbapenemase in most countries, and its rapid spread threatens global public health. *bla*_KPC_ transmission is commonly mediated by Tn*4401* transposons. The *bla*_KPC_ gene has also been found in *non-Tn4401 elements* (NTE_KPC_). To fill the gap in the understanding of the stability and dissemination of NTE_KPC_-carrying plasmids, we selected and characterized carbapenem-resistant bacteria isolated between 2009 and 2016 from a hospital for a retrospective study of their plasmids conjugation capacity, impact on fitness, and replication in different species. Different clones were selected using PFGE, and their genomes were sequenced using Illumina and Oxford Nanopore methods. Minimum inhibitory concentrations (MICs) were determined by broth microdilution. Plasmid copy numbers (PCNs) were determined using qPCR. Doubling time was used to analyze fitness change. Most isolates (67%, 33/49) carried *bla*_KPC_, of which 85% presented *bla*_KPC_ in a NTE_KPC_. The 25 isolates selected presented the *bla*_KPC_ gene in NTE_KPC_-IId in IncQ1-type plasmids, showing multispecies dissemination. IncQ1 plasmids were mobilizable and PCN seemed to be directly linked to the species, presenting a high-copy number, mainly in *K. pneumoniae*. No relationship was observed between IncQ1 PCN and carbapenems MIC values. IncQ1 and a conjugative plasmid from *K. pneumoniae* BHKPC10 were transferred to *E. coli* J53 without fitness changes, and MIC values were maintained for carbapenems despite the low transconjugant PCN. In addition to IncQ1 with NTE_KPC_, *Enterobacter cloacae* BHKPC28 contained the *mcr-9* gene in an IncHI2/IncHI2A conjugative plasmid, which may help the mobility of IncQ1 and the dissemination of two resistance determinants to last-resort antibiotics. Understanding the interaction between plasmids and high-risk lineages can help develop new therapies to prevent the dissemination of resistance traits.

## 1. Introduction

Carbapenemases are enzymes with a broad spectrum for degrading β-lactams, conferring resistance to carbapenem antibiotics despite having the potential to hydrolyze practically all β-lactams [1,2,3,4].

Gram-negative bacteria have different serine carbapenemases; however, KPC, encoded by the *bla*_KPC_ gene, has gained worldwide attention. KPC was first identified in 1996 in a *Klebsiella pneumoniae* isolate from North Carolina, USA, on a non-conjugative plasmid [2,5,6,7]. KPC enzymes are Ambler class A β-lactamases comprising approximately 90 variants, with a predominance of KPC-2 and KPC-3 [8,9,10]. Currently, KPC is the most clinically significant serine carbapenemase in Brazil, the United States, and most countries worldwide, and its rapid international spread has threatened global public health [11,12,13]. KPC spread has been related to *K. pneumoniae* high-risk sequence types (STs), mainly ST258 and a single-locus variant ST512 [14,15,16]. However, in some European countries, the *K. pneumoniae* KPC-carrying epidemiology has changed, and ST258 is not the most prevalent clone [15,16]. *K. pneumoniae* high-risk lineage ST11 is mostly reported among *bla*_KPC_-harboring *K. pneumoniae* isolates in Asia, especially China, and seems to have replaced ST258 in the Balkan Peninsula [15,17]. Another high-risk clonal lineage to highlight is ST147, which has already been described in Greece, Spain, and France as carrying *bla*_KPC_ [15,16].

The *bla*_KPC_ gene was initially associated with Tn*4401*, a Tn3-type transposon [18,19,20]. In addition to the *bla*_KPC_ gene, Tn*4401* has a gene for a transposase (*tnpA*), a resolvase (*tnpR*), and two insertion sequences (IS) (IS*Kpn6* and IS*Kpn7*). Although most *bla*_KPC_ genes are present in Tn*4401*, they have been found in other genetic environments, referred to as non-Tn*4401* element-carrying *bla*_KPC_ (NTE_KPC_) [11,18,21]. Studies conducted in different countries show different NTE_KPC_ being detected worldwide associated with *bla*_KPC_ [22,23,24,25,26]. The impact of changing the genetic environment on the stability and dissemination of this resistance gene remains unknown. As *bla*_KPC_ was commonly detected in Tn*4401*, Brazilian studies rarely carried out surveillance of the dissemination of the gene in other genetic environments [27,28,29,30].

Mobile genetic elements (EGMs) such as integrons, insertion sequences, transposons, and mobile plasmids enable the dissemination of resistance genes between bacteria of the same or different species, by horizontal transfer. Plasmids play a crucial role in bacterial adaptation and survival in unsuitable environments and directly impact the control of antibiotic resistance, as they can be transferred [2,31,32]. The genetic environment Tn*4401* and its isoforms and the conjugative/mobilizable plasmids carrying this transposon confer mobility to the *bla*_KPC_ gene [18,33,34]. Conjugative plasmids present a type IV secretion system (T4SS) to form the conjugation channel and a mobility module (MOB), which includes an origin of transfer (*oriT*), a relaxase, and a type IV coupling protein. However, plasmids producing only one *oriT* and one relaxase are mobilizable because they can sequester the T4SS systems encoded by other mobile genetic elements within the same bacterial cell [35].

These plasmids may contain genes that confer resistance to other antimicrobial agents, such as aminoglycosides, quinolones, trimethoprim, sulfonamides, and tetracyclines. These characteristics increase the complexity of controlling the spread of these plasmids as co-selection leads to the transmission of multidrug resistance [11].

In this study, we molecularly characterized clinical isolates harboring *bla*_KPC_ in NTE_KPC_-IId from a teaching hospital in Brazil’s southeast. We analyzed the plasmid types carrying this genetic environment in terms of their conjugation capacity, impact on fitness, and replication in different species. We demonstrated the presence of an IncQ1 plasmid NTE_KPC_-carrying that disseminated to different high-risk clones of different species at the same hospital. We also analyzed the dissemination of this plasmid, showing what happens naturally in the hospital environment, as demonstrated in vitro by Martins et al., 2020 [36].

## 2. Materials and Methods

### 2.1. Bacterial Isolates

A total of 49 carbapenem-resistant *Enterobacterales* (CRE) isolates collected between 2009 and 2016 at a teaching hospital in Belo Horizonte, southeast Brazil, were considered for this study. The isolates belonged to different species, had different resistance profiles, and were isolated from patients hospitalized at various periods. Clinical specimens included axillary swab, blood, urine, sacral eschar swab, calcaneal tendon, tracheal aspirate, mini-bronchoalveolar lavage, deep tissue, and abdominal abscess, without restrictions on the colonization or infection site. All bacterial isolates were registered at SISGen under the number AAE0048. The study was analyzed by the FCFRP-USP ethical committee and the National Health Council (CONEP) (CAAE: 51267321.4.0000.5403).

*E. coli* J53 strain was selected as the recipient for the conjugation assays. *K. pneumoniae* ATCC BAA-1705 and *K. pneumoniae* ATCC BAA-1706 strains were used as controls for PCR reactions. *E. coli* ATCC 25922 was used as a control to determine the minimal inhibitory concentration using the broth microdilution method.

For bacterial cultures, the isolates were cultivated in a BHI medium (Kasvi, Parana, Brazil) and grown in an incubator without shaking at 37 °C.

### 2.2. Molecular Assays

Susceptibility to imipenem (Goldbio, St. Louis, MO, USA), meropenem (ABL—Antibióticos do Brasil, Cosmópolis, SP, Brazil), colistin (Sigma Aldrich, Saint Louis, MO, USA), and polymyxin B (Sigma Aldrich, Saint Louis, MO, USA) was determined using the broth microdilution method according to ISO 20776-1 [37]. Genomic DNA was extracted as described by Boralli et al. (2023) [38]. The quality and quantity of the DNA obtained after extraction were measured using a NanoDrop^®^ 2000 spectrophotometer (Thermo Fisher Scientific, Waltham, MA, USA). The *bla*_KPC_ gene and Tn*4401* detection by PCR was performed using primers described by Poirel et al. (2011) and Naas et al. (2008) under the conditions already defined by our group [18,38,39]. Clonality was evaluated using pulsed-field gel electrophoresis after macrorestrition with XbaI (New England Biolabs, Ipswich, MA, USA) in 1% Seakem Gold Agarose (Lonza, Basel, Switzerland). The run conditions were a 6 V gradient, 19 h, 6.76 s initial switch time, and 35.35 s final switch time using the CHEF Mapper system (Bio-Rad, Hercules, CA, USA). The gels were dyed with SYBR Safe DNA Gel Stain (Thermo Fisher Scientific, Waltham, MA, USA) and revealed using the ChemiDoc MP Imaging System (Bio-Rad, Hercules, CA, USA). The PFGE gel image was analyzed using Bionumerics software v. 7.1 (Applied Maths, Sint-Martens-Latem, Belgium).

Plasmid size detection was performed according to the method described by Boralli, et al., 2023 [38].

### 2.3. Genome Sequencing

Genome sequencing was performed using Illumina and Nanopore technologies at the University Medical Center Utrecht (The Netherlands). For Illumina assays, the Nextera DNA sample preparation kit and Nextera index v2 set D for 96 indexes were used to prepare the library, and NextSeq500 2 × 150 bp mid-output (120 M clusters) (Illumina, San Diego, CA, USA) was used for sequencing [38].

For nanopore sequencing, Oxford Nanopore’s Ligation Sequencing Kit and Native Barcoding Expansion Kit were used for library preparation, and the MinION sequencer (Oxford Nanopore Technologies, Oxford, UK) was used for the sequencing in flow cells [38].

### 2.4. Quality Analysis, Genome Assembly, and Functional Analysis

Fastqc (v0.11.9) was used for the initial quality analysis of Illumina reads. Adapter removal and quality trimming analyses were performed using Trim Galore (v.0.6.6) (https://github.com/FelixKrueger/TrimGalore, accessed on 1 February 2023) with default parameters. Genomes were assembled using Unicycler [40] (v.0.4.8) in bold mode. BUSCO [41] (v5.1.2) software was used to assess the quality of the hybrid assemblies.

The assembled genomes were submitted to the NCBI database for annotation and deposited with accession numbers presented in Table S2.

Genome sequences were analyzed using ResFinder to predict the resistance gene content, MLST to infer sequence types, and VirulenceFinder to identify virulence genes. All tools, available at the Center for Genomic Epidemiology (http://www.genomicepidemiology.org/, accessed on 8 March 2023), were used with the default parameters.

### 2.5. Plasmids Analyzes

Conjugation assays between the receptor strain *Escherichia coli* J53 and the donor strains were performed as described by Boralli et al. (2023), as well as growth curve analysis, doubling time determination, and plasmid copy number determination with specific primers for each species (Table S1). Plascad software v0.7.8 was used with default parameters to predict plasmid mobilization capacity with default parameters [38].

## 3. Results

### 3.1. The Bla_kpc_ Gene and NTE_KPC_ Occurrence in the Bacterial Isolates

Forty-nine *Enterobacterales* isolates belonging to different species, resistant to at least one carbapenem, were initially analyzed in this study (Table 1). We screened them for the presence of the *bla*_KPC_ gene in NTE_KPC_ and found an occurrence of 67% (33/49) of bacterial isolates carrying *bla*_KPC_, of which 85% (28/33) presented *bla*_KPC_ in a NTE_KPC_ genetic environment. Out of 18, 5 (27.8%) *K. pneumoniae* had *bla*_KPC_ in Tn*4401*. Additionally, to ensure high genetic diversity, three *K. pneumoniae* isolates were excluded from this study due to 100% genetic similarity by PFGE, indicating that they were the same clone (Figure S1).

Twenty-five of these Enterobacterales isolates were selected for sequencing. Quality analysis results indicated high-sequencing coverage, with an average depth of approximately 100×, demonstrating reliable data (Table S2). All genomes sequenced were submitted to the NCBI, and accession numbers are given in Table S2.

### 3.2. NTE_KPC_ and Bla_kpc_-Carrying Plasmids Analysis in the Sequenced Isolates

The sequenced isolates belonged to 15 known STs, indicating significant genetic diversity (Table 2). All *Providencia stuartii* isolates presented unknown MLST profiles. Additionally, all the strains presented *bla*_KPC_ in the same genetic environment, NTE_KPC_-IId, which harbored the following genes in addition to *bla*_KPC_: *tnpR*, Δ*bla*_TEM_, and *ISKpn6*.

**Figure 1:**
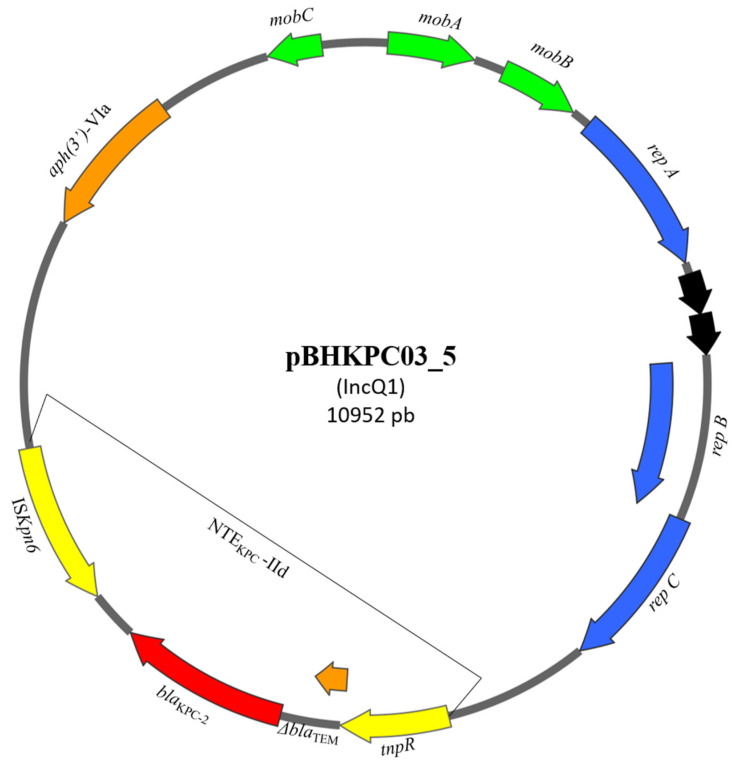
Scheme of the BHKPC03 IncQ1 plasmid harboring *bla*_KPC_ in NTE_KPC_-IId. *bla*_KPC_ gene is shown in red. Other resistance genes are shown in orange. NTE_KPC_-IId genes are shown in yellow. Genes related to plasmid mobility capacity are shown in green. Replication genes are shown in blue.

NTE_KPC_-IId transposon was harbored by IncQ1 plasmids. IncQ1 plasmids found in this study ranged from 10941 to 18248 bp (Figure 1 and Table 2) and harbored another resistance gene, in addition to NTE_KPC_-IId. The *aph(3*′*)*-Via gene encodes an aminoglycoside phosphotransferase related to aminoglycoside resistance. Furthermore, IncQ1 plasmids are classified as non-conjugative but mobilizable, as they depend on a T4SS of a conjugative plasmid for horizontal transfer.

**Figure 2:**
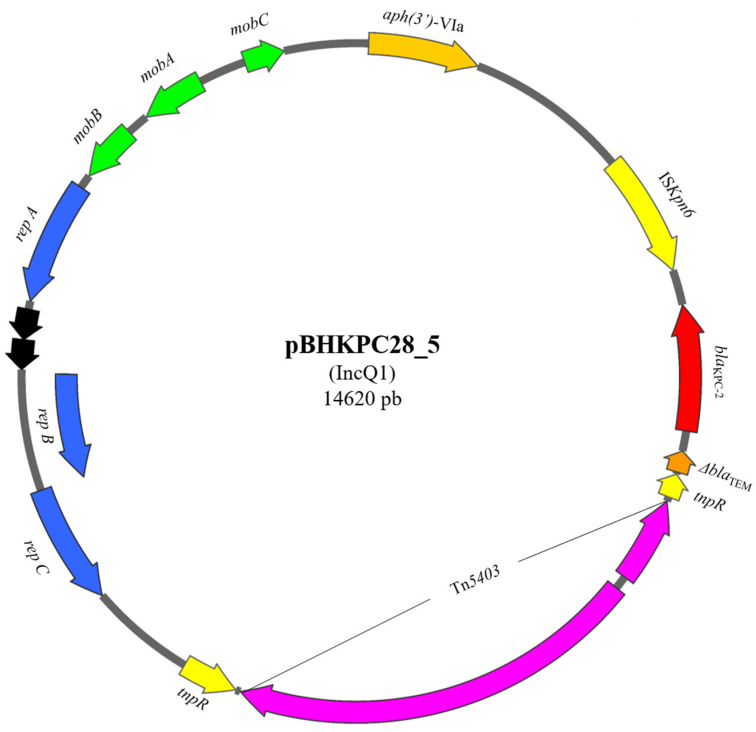
Scheme of the pBHKPC28_5 plasmid harboring *bla*_KPC_ gene. *bla*_KPC_ is presented in red. Other resistance genes are shown in orange. NTE_KPC_-IId genes are shown in yellow. Genes related to the plasmid mobility capacity are shown in green. Replication genes are shown in blue. The transposon inserted into NTE_KPC_-IId is indicated in pink.

*E. cloacae* BHKPC28 presented a 14 kbp IncQ1 plasmid due to an insertion of a Tn*5403* transposon (Figure 2), and *K. aerogenes* BHKPC52 showed an IncQ1 plasmid with a duplication in the region that was not part of NTE_KPC_-IId (Figure S2).

### 3.3. Plasmid Populations in the Sequenced Isolates

Although the plasmids harboring the *bla*_KPC_ gene were similar among most isolates, different plasmid populations were observed in each isolate. These plasmids present different characteristics related to the incompatibility group, conjugation/mobilization capacity, and harbored resistance genes (Tables S3–S7).

Eight isolates from three species, two *E. coli*, one *K. pneumoniae*, and five *P. stuartii*, contained only the IncQ1 plasmid in their genomes. Furthermore, only *K. pneumoniae* BHKPC44 and *E. hormaechei* BHKPC07 did not contain any conjugative plasmid (Tables S3 and S5–S7).

Among the *K. pneumoniae* isolates, we observed some plasmid population diversity, although groups with similar plasmid populations were observed, such as BHKPC03, BHKPC04, and BHKPC18, in addition to BHKPC08 and BHKPC10 (Table 3).

*K. aerogenes*, *E. hormaechei*, and *E. cloacae* isolates showed the diversity of plasmids, and none presented only the *bla*_KPC_-carrying plasmid. *K. aerogenes* BHKPC06 and BHKPC52 harbored similar plasmid populations, except for the IncM1 plasmid presence in *K. aerogenes* BHKPC06 and the differences in mobilization and resistance genes present in the IncC plasmids (Tables S4 and S5).

*E. coli* isolates presented a diversity of plasmids, with IncQ1 being the only plasmid shared among the strains. *E. coli* BHKPC11 and BHKPC13 were the only isolates that harbored only the IncQ1 plasmid, although they harbored other resistance genes on their chromosomes (Table S6).

*P. stuartii* isolates had the lowest plasmid population diversity, with five of the seven isolates harboring the IncQ1 plasmid alone. Furthermore, the chromosomes of the seven isolates harbored the same resistance genes: *aac(2*′*)-Ia*, *catA3*, and *tet(B)*. Finally, *P. stuartii* BHKPC27 and BHKPC35 harbored two other conjugative plasmids, including an IncC plasmid harboring eight resistance genes (Table S7).

### 3.4. Carbapenems MIC and PCN Analysis According to Plasmid Population and Species

We did not observe a high diversity of genetic environments or plasmids carrying *bla*_KPC_. However, there are isolates with different plasmid populations and from various species that harbor the *bla*_KPC_ gene.

To analyze whether different backgrounds can influence the host’s carbapenems resistance phenotype and IncQ1 plasmid replication, the MIC values of imipenem and meropenem and the PCNs were compared among the isolates (Table 3).

Carbapenem MIC values were similar among the isolates, except for *K. aerogenes* BHKPC06 and BHKPC52, which presented higher values than the other isolates. Notably, the two isolates belonged to the same species and had similar plasmid populations (Table 3).

All *P. stuartii* isolates exhibited high MIC values for imipenem. *E. coli* BHKPC37 presented MIC values for carbapenems that classified them as susceptible, according to the parameters defined by EUCAST (Table 3).

The data indicated a correlation between PCN and the species harboring the *bla*_KPC_ gene. The highest values were found in *K. pneumoniae* isolates, except *K. pneumoniae* BHKPC15, the only isolate of this species harboring only the IncQ1 plasmid carrying *bla*_KPC_ (Table 3 and Table S3). *K. pneumoniae* BHKPC15 was the only *K. pneumoniae* isolate that harbored only the IncQ1 plasmid carrying *bla*_KPC_ (Table 3 and Table S3).

The lowest PCN values were obtained for *P. stuartii* isolates, regardless of the plasmid population, indicating that IncQ1 plasmids were present in a single copy (Table 3). Isolates of *E. coli*, *K. aerogenes*, and *Enterobacter* spp. presented similar PCN values (Table 3).

No patterns were observed between MIC values and PCN.

### 3.5. Analysis of Plasmids Carrying Bla_kpc_ Transfer

*K. pneumoniae* isolates that harbored the IncQ1 plasmid carrying *bla*_KPC_ and presented different plasmid populations were selected for conjugation assays. Since IncQ1 plasmids carrying *bla*_KPC_ were classified as mobilizable and non-conjugative, it would not be possible to attempt to conjugate it from strains that only carry it or carry it with other non-conjugative plasmids. *K. pneumoniae* BHKPC03, BHKPC04, BHKPC08, BHKPC10, BHKPC18, BHKPC21, BHKPC47, and BHKPC50 were selected, and a transconjugant was obtained from the assay using *K. pneumoniae* BHKPC10 as a donor strain with a conjugation rate of 1.49 × 10^−4^.

From the analysis of the S1 nuclease gel, it was possible to confirm the transfer of a plasmid of approximately 10 kbp and two other plasmids that were approximately 40–50 kbp. The pBHKPC10_2 plasmid was approximately 50 kbp and was conjugative, indicating that it enabled the transfer of pBHKPC10_3 during its conjugation. However, genome sequencing data for *K. pneumoniae* BHKPC10 showed the presence of only one plasmid measuring approximately 40–50 kbp. The second plasmid, with a size of 40–50 kbp, present in *K. pneumoniae* BHKPC10 and its transconjugant may indicate that there are similar plasmids in sequences that were mistakenly grouped during the assembly of the genome into a larger plasmid (Figure S3).

The doubling time showed no change in the fitness of the *E. coli* J53 strain after receiving the described plasmids, indicating that the presence of these plasmids did not cause significant energy costs for their persistence (Table 4).

The MIC values for imipenem and meropenem differed between species, with slightly higher values for *K. pneumoniae* BHKPC10 and its transconjugant *E. coli* J53_pBHKPC10_2_3. Although the transconjugant presented lower MIC values, there was a significant increase in the MIC compared to *E. coli* J53 before conjugation. Furthermore, relative PCN determination assays showed that the copy number of this plasmid was approximately 70 times higher in *K. pneumoniae* BHKPC10 than in *E. coli* J53_pBHKPC10_2_3 (Table 4).

### 3.6. Description of a Strain Carrying Bla_kpc_ and Mcr-9 Genes

*E. cloacae* BHKPC28 belongs to ST850 isolated in 2014 and was the only isolate in this study that in addition to the *bla*_KPC_ gene, harbors the *mcr-9* gene. The *mcr-9* gene encodes a phosphoethanolamine transferase responsible for modifying lipid A of lipopolysaccharide (LPS) conferring resistance to polymyxins, drugs also considered as the last resource [42]. This isolate had colistin and polymyxin B MIC values of 16 and 8 mg/L, respectively, and was considered resistant according to EUCAST.

In addition to the *mcr-9* gene, *E. cloacae* BHKPC28 harbors 26 resistance genes distributed on its chromosome and on four of its eight plasmids, two of which are conjugative, one mobilizable, and one non-conjugative. The eight plasmids had sizes ranging from 3.6 to 285 kbp and belonged to six different incompatibility groups; pBHKPC28_6 and pBHKPC28_7 could not be typed (Table 5).

The pBHKPC28_5 plasmid was 4 kbp larger than most of the other IncQ1 plasmids because of the insertion of the Tn*5403* transposon in the *tnpR* gene of NTE_KPC_-IId (Figure 2). This change in the genetic environment did not result in different MIC values compared with those found for other isolates of the same species that harbored the intact NTEKPC-IId. The relative NCP value of pBHKPC28_5 was close to the values found for IncQ1 plasmids harboring *bla*_KPC_ from isolates of the same species, indicating that the insertion of Tn*5403* did not alter plasmid replication (Table 6).

pBHKPC28_1 is a conjugative IncHI2/IncHI2A plasmid of approximately 285 kbp that harbors the *mcr-9* gene and 12 other resistance genes: *aac(6*′*)-Ib-cr*, *aph(3*″*)-Ib*, *sul1*, *sul2*, *dfrA19*, *qnrA1*, *tet(A)*, *bla_OXA-1_*, *bla_CTX-M-15_*, *bla_TEM-1B_*, *qacE* e *catB3*.

**Figure 3:**
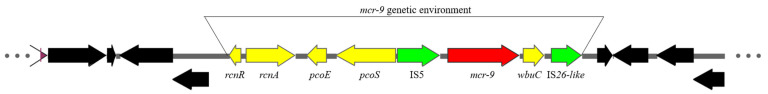
Scheme of *mcr-9* genetic environment of the pBHKPC28_1 plasmid. *mcr-9* gene is indicated in red. *mcr-9* genetic environment is shown in yellow, and IS genes are shown in green.

In this plasmid, the *mcr-9* gene was found in a genetic environment containing the *rcnR*, *rcnA*, *pcoE*, *pcoS*, and *wbuC* genes in addition to the two IS (IS5 and IS26-like) that flank the gene (Figure 3).

## 4. Discussion

Currently, KPC is the most clinically significant serine carbapenemase in Brazil, the United States, and most countries worldwide, and its rapid international spread threatens global public health [11,12,13,43]. This study found that the *bla*_KPC_ gene was associated with NTE_KPC_ in high-risk clonal lineages such as *K. pneumoniae* ST11, which seems to be replacing ST258 as the most prevalent clone in Europe [15,16]. The high-risk clonal lineages *K. pneumoniae* ST147 and *E. coli* ST127 were also detected carrying the *bla*_KPC_ gene in this study [16,44].

The transmission of the gene that encodes KPC, *bla*_KPC_, can be mediated by different molecular mechanisms, from the mobility of small genetic elements to the horizontal transfer of plasmids and via clonal dissemination [11,45]. This gene was originally found associated with Tn*4401*, a Tn3-type transposon, and this genetic environment and its isoforms are attributed to the mobility of the gene, as well as to the plasmids where this transposon is found [18,20,33,34]. However, this gene has been found in other genetic environments, referred to as NTE_KPC_, and the impact of changing the genetic environment on the stability and dissemination of this resistance gene remains unknown [11,18,21]. As *bla*_KPC_ is commonly detected in Tn*4401*, surveillance of gene dissemination in other genetic environments has rarely been conducted in Brazilian studies [27,29,30].

Thirty-three isolates carrying *bla*_KPC_ were found among the 49 strains initially selected, resulting in an occurrence of approximately 67%. The remaining 16 isolates that lacked the *bla*_KPC_ gene might have genes coding for other carbapenemases, such as *bla*_NDM_, or even other resistance mechanisms, such as overexpression of efflux pumps, modifications in porins, or target enzymes of antibiotics of this class. This rate was expected because KPC expression is the most common mechanism of carbapenem resistance in Brazil [43,46].

*K. pneumoniae* species predominated among the isolates carrying the *bla*_KPC_ gene (18/33), which is consistent with the literature. A higher incidence of *bla*_KPC_ has been reported in *K. pneumoniae*, the species in which this carbapenemase was identified for the first time, despite its spread to different species of Gram-negative bacteria [2,5,6,7,11].

The *bla*_KPC_ genetic environment analysis of the selected isolates showed a Tn*4401* occurrence of approximately 15% (5/33). This profile was different from what was expected since the Tn*4401* transposon and its isoforms were the most responsible for the spread of the *bla*_KPC_ gene [18,33,34].

Despite the genetic diversity indicated by the STs of the 25 sequenced isolates, there was little diversity in the *bla*_KPC_ gene genetic context (NTE_KPC_-IId) without significant changes in all isolates, except *E. cloacae* BHKPC28. Furthermore, this genetic environment was found in similar mobilizable IncQ1 plasmids in all the isolates. These data indicated the dissemination of the IncQ1 plasmid carrying *bla*_KPC_ in this hospital between 2009 and 2016. IncQ1 plasmids harboring NTE_KPC_-IId are known nationally and have been described in different regions of Brazil [47,48,49,50,51].

IncQ1 plasmids have a mobilization module and depend on the T4SS of other plasmids for their transfer. While conjugative plasmids generally have a low copy number, small mobilizable plasmids tend to have high copy numbers [49,52].

Isolates presenting only mobilizable or non-mobilizable plasmids in their genome are unable to transfer these plasmids horizontally to other bacteria due to the absence of a T4SS. Evolutionarily, isolates like these must have had a conjugative plasmid to enable the non-conjugative plasmid transfer. IncQ1 plasmids carrying NTE_KPC_-IId found in this study spread to different species, indicating horizontal gene transfer. However, as the IncQ1 plasmids were classified as mobilizable, conjugative plasmids probably previously enabled their conjugation and were lost by the bacteria.

The presence of IncQ1 plasmids in different species carrying other plasmids made it possible to analyze them in various contexts.

Although no direct relationship was observed between PCN and carbapenem MICs in the analyzed isolates, the data indicated a relationship between the PCN and the species harboring the *bla*_KPC_ gene. The lowest values were observed in *P. stuartii* isolates, indicating that IncQ1 plasmids were present in a single copy. These isolates also had the lowest plasmid population diversity, with few resistance genes distributed on a few plasmids, compared to most of the other isolates. The imipenem MICs for this species were high, but EUCAST warned about the intrinsically low activity of imipenem against *Morganella morganii*, *Proteus* spp., and *Providencia* spp. The highest PCN values were observed in *K. pneumoniae* isolates, except for BHKPC15, whose plasmid population was composed of only the *bla*_KPC_-carrying plasmid.

Martins et al., 2020 estimated 20 copies of an IncQ1 plasmid containing NTE_KPC_-Ic, in *K. pneumoniae*. The copy numbers found in this study for *K. pneumoniae*, according to the methodology used, were higher than those in the cited study. Despite having obtained transconjugants, the PCN was not estimated in another species by Martins et al. 2020 to determine if there was a difference between the species. The PCN difference found in this study might be a reason for the higher incidence of the *bla*_KPC_ gene in *K. pneumoniae* [2,36].

Conjugation of the IncQ1 plasmid to *E. coli* J53 was successful only when *K. pneumoniae* BHKPC10 was used as the donor strain. The high PCN of pBHKPC10_3 and the conjugative plasmid pBHKPC10_2 may have contributed to the transfer of pBHKPC10_3. The conjugated plasmids did not induce changes in the bacteria’s fitness and significantly increased carbapenems MICs in the transconjugant strain, contributing to their persistence in the cell. Once again, a pattern of PCN and species harboring the plasmid was observed, as the PCN in *E. coli* was comparatively lower than that of the same plasmid in *K. pneumoniae*. These data corroborate the literature that classifies IncQ1 plasmids as small replicons known for their stability, low impact on bacterial fitness, and high mobilization potential, regardless of whether they are non-conjugative [47,49,50].

Finally, this study detected the *E. cloacae* BHKPC28 isolate harboring the *mcr-9* gene, in addition to *bla*_KPC_. Unlike the *mcr-9* variant described by Martins et al. (2021) and Kieffer et al. (2019), *E. cloacae* BHKPC28 had a *mcr-9* gene variant and the polymyxins-resistance phenotype [53,54]. The *mcr-9* gene was on an IncHI2/IncHI2A plasmid and its genetic environment is composed of the *rcnR* gene, which encodes a repressor of the *rcnA* gene, a nickel and cobalt efflux pump, in addition to *pcoE* and *pcoS*, involved in copper tolerance. The presence of these genes suggests that the strain is also tolerant to heavy metals [55,56]. Heavy metal compounds can be used to treat bacterial infections. The presence of heavy metals tolerance mechanisms may enable the bacteria to survive under extremely unfavorable conditions when co-selected with antibiotic resistance mechanisms [57]. IncHI2IncHI2A plasmids have already been linked to the spread of the *mcr-9* gene as well as the genetic environment harboring the gene [57,58,59,60]. The *E. hormaechei* BHKPC43 isolate harbored an IncHI2/IncHI2A plasmid similar in size to pBHKPC28_1, and both shared 99.98% identity and 76% coverage, indicating that this plasmid might have lost the *mcr-9* gene and continued to spread throughout the hospital, leading to a co-selection of heavy metal tolerance and antibiotics resistance.

Furthermore, in BHKPC28, there was a Tn*5403* insertion in the *tnpR* gene of NTE_KPC_ harbored in pBHKPC28_5, but this change kept the region responsible for plasmid replication intact. Therefore, the PCN values obtained were similar to those of the other IncQ1 plasmids of the same species isolates. Despite this insertion into the genetic environment harboring *bla*_KPC_, NTE_KPC_ was not directly modified, and carbapenem MICs were similar to those of the same species isolates. Finally, one of the major concerns regarding this isolate was that, as pBHKPC28_1 was a conjugative plasmid, it could enable the transfer of pBHKPC28_5 during its conjugation process and then disseminate two resistance genes to antibiotics considered as a last resort, in addition to resistance to several other antibiotics and heavy metals tolerance, further limiting the possibility of treating infections caused by bacteria.

## 5. Conclusions

This study showed multispecies dissemination of IncQ1-type plasmids carrying the *bla*_KPC_ gene in NTE_KPC_-IId between 2009 and 2016 in a Brazilian hospital. The isolates belonged to different STs, had different plasmid populations, and had varied MIC values for carbapenems. The species harboring the IncQ1 plasmid influenced its copy number, with the highest values observed in *K. pneumoniae*. This behavior was also observed when comparing the same plasmid conjugated from this species to *E. coli*, without significant fitness changes. The dissemination of this plasmid is a threat to public health and becomes even more dangerous when associated with plasmids carrying other resistance genes to different last-resort antibiotics, as observed for *mcr-9* in the BHKPC28 isolate. This study added information about the dissemination of IncQ1 plasmids, which must be closely monitored, as they are directly related to the dissemination of the *bla*_KPC_ gene. Understanding the interaction between plasmids and high-risk lineages can help develop new therapies to prevent the dissemination of resistance traits.

## Figures and Tables

**Table 1:** Number of NTE_KPC_-carrying isolates out of the total isolates carrying *bla*_KPC_ in each species.

*K. pneumoniae*		*P. stuartii*		*K. aerogenes*		*Enterobacter* spp.		*E. coli*	
*bla*_KPC_	NTE_KPC_	*bla*_KPC_	NTE_KPC_	*bla*_KPC_	NTE_KPC_	*bla*_KPC_	NTE_KPC_	*bla*_KPC_	NTE_KPC_
18	13	7	7	2	2	3	3	3	3

**Table 2:** Sequence type, genetic environment, and plasmids of *bla*_KPC_ of sequenced isolates.

Species	Isolate	ST	*bla*_KPC_ Genetic Environment	*bla*_KPC_ Plasmid	*bla*_KPC_ Plasmid Sizes (bp)
*K. pneumoniae*	BHKPC03	11	NTE_KPC_-IId	IncQ1	10,946
*K. pneumoniae*	BHKPC04	11	NTE_KPC_-IId	IncQ1	10,952
*K. aerogenes*	BHKPC06	4	NTE_KPC_-IId	IncQ1	10,948
*E. hormaechei*	BHKPC07	93	NTE_KPC_-IId	IncQ1	10,949
*K. pneumoniae*	BHKPC08	35	NTE_KPC_-IId	IncQ1	10,941
*K. pneumoniae*	BHKPC10	35	NTE_KPC_-IId	IncQ1	10,947
*E. coli*	BHKPC11	1001	NTE_KPC_-IId	IncQ1	10,952
*E. coli*	BHKPC13	127	NTE_KPC_-IId	IncQ1	10,982
*K. pneumoniae*	BHKPC15	603	NTE_KPC_-IId	IncQ1	10,949
*K. pneumoniae*	BHKPC18	11	NTE_KPC_-IId	IncQ1	10,950
*K. pneumoniae*	BHKPC21	7946	NTE_KPC_-IId	IncQ1	10,946
*P. stuartii*	BHKPC23	-	NTE_KPC_-IId	IncQ1	10,949
*P. stuartii*	BHKPC27	-	NTE_KPC_-IId	IncQ1	10,946
*E. cloacae*	BHKPC28	850	NTE_KPC_-IId	IncQ1	14,620
*P. stuartii*	BHKPC29	-	NTE_KPC_-IId	IncQ1	10,949
*P. stuartii*	BHKPC30	-	NTE_KPC_-IId	IncQ1	10,948
*P. stuartii*	BHKPC31	-	NTE_KPC_-IId	IncQ1	10,949
*P. stuartii*	BHKPC35	-	NTE_KPC_-IId	IncQ1	10,949
*E. coli*	BHKPC37	457	NTE_KPC_-IId	IncQ1	10,950
*P. stuartii*	BHKPC41	-	NTE_KPC_-IId	IncQ1	10,949
*E. hormaechei*	BHKPC43	114	NTE_KPC_-IId	IncQ1	10,948
*K. pneumoniae*	BHKPC44	29	NTE_KPC_-IId	IncQ1	10,948
*K. pneumoniae*	BHKPC47	147	NTE_KPC_-IId	IncQ1	10,952
*K. pneumoniae*	BHKPC50	874	NTE_KPC_-IId	IncQ1	10,949
*K. aerogenes*	BHKPC52	4	NTE_KPC_-IId	IncQ1	18,248

**Table 3:** MIC of imipenem and meropenem and relative PCN of the plasmids carrying *bla*_KPC_ of sequenced isolates.

Species	Isolates	MIC_Imipenem_ (mg/L)	MIC_Meropenem_ (mg/L)	*bla*_KPC_ Plasmids PCN
*K. pneumoniae*	BHKPC03	8	16	99 ± 1
*K. pneumoniae*	BHKPC04	32	32	417 ± 4
*K. pneumoniae*	BHKPC08	32	32	125 ± 2
*K. pneumoniae*	BHKPC10	32	32	357 ± 4
*K. pneumoniae*	BHKPC15	16	32	18.8 ± 0.8
*K. pneumoniae*	BHKPC18	64	64	152 ± 1
*K. pneumoniae*	BHKPC21	32	32	223 ± 2
*K. pneumoniae*	BHKPC44	8	16	109.3 ± 0.7
*K. pneumoniae*	BHKPC47	8	16	110 ± 3
*K. pneumoniae*	BHKPC50	8	16	335 ± 3
*K. aerogenes*	BHKPC06	64	128	12.9 ± 0.2
*K. aerogenes*	BHKPC52	64	256	17.7 ± 0.8
*E. hormaechei*	BHKPC07	8	16	16.4 ± 0.3
*E. cloacae*	BHKPC28	8	16	23 ± 0.5
*E. hormaechei*	BHKPC43	32	64	16 ± 1
*E. coli*	BHKPC11	8	8	20.7 ± 0.9
*E. coli*	BHKPC13	8	16	22.5 ± 0.7
*E. coli*	BHKPC37	4	4	22.8 ± 0.5
*P. stuartii*	BHKPC23	128	16	0.43 ± 0.02
*P. stuartii*	BHKPC27	128	32	0.94 ± 0.5
*P. stuartii*	BHKPC29	64	16	0.73 ± 0.02
*P. stuartii*	BHKPC30	128	32	0.90 ± 0.08
*P. stuartii*	BHKPC31	64	16	1.27 ± 0.08
*P. stuartii*	BHKPC35	128	32	1.25 ± 0.07
*P. stuartii*	BHKPC41	64	16	0.93 ± 0.06

**Table 4:** Doubling time, MIC, and PCN analysis of *K. pneumoniae* BHKPC10 plasmids transfer.

	Doubling Time (min)	MIC_Imipenem_ (mg/L)	MIC_Meropenem_ (mg/L)	PCN pBHKPC10_3
*K. pneumoniae* BHKPC10		32	32	358 ± 5
*E. coli* J53	117 ± 4	0.125	0.0625	-
J53_pBHKPC10_2_3	121 ± 5	8	16	5.1 ± 0.2

**Table 5:** Data of the resistance gene’s location and characterization of plasmid populations present in *E. cloacae* BHKPC28 isolate.

Chromosome/Plasmids	Contig	Classification	Resistance Genes	Mobilization	Size (bp)
Chromosome_BHKPC28	1	-	*fosA*, *blaCMH-3*	-	5,025,881
pBHKPC28_1	2	IncHI2/IncHI2A	*mcr-9*, *aac(6*′*)-Ib-cr*, *aph(3*″*)-Ib*, *sul1*, *sul2*, *dfrA19*, *qnrA1*, *tet(A)*, *bla_OXA-1_*, *bla_CTX-M-15_*, *bla_TEM-1B_*, *qacE*, *catB3*	Conjugative	284,726
pBHKPC28_2	3	IncFIIY	*-*	Conjugative	110,191
pBHKPC28_3	4	IncFII	*-*	Conjugative	64,936
pBHKPC28_4	5	pBSSB1-family	*aadA1*, *aac(6*′*)-Ib-cr*, *aac(6*′*)-Ib*, *bla_OXA-9_*, *bla_TEM-1A_*	Conjugative	53,873
pBHKPC28_5	6	IncQ1	*aph(3*′*)-*Via, *bla_KPC-2_*	Mobilizable	14,620
pBHKPC28_6	8	NI	*-*	Mobilizable	5560
pBHKPC28_7	9	NI	*-*	Mobilizable	3869
pBHKPC28_8	10	IncN	*aac(6*′*)-Ib-cr*, *aac(6*′*)-Ib3*, *bla_OXA-1_*, *catB3*	Not mobilizable	3692

**Table 6:** Carbapenems MIC and PCN values of BHKPC28.

	MIC_Imipenem_ (mg/L)	MIC_Meropenem_ (mg/L)	PCN pBHKPC28_5
*E. cloacae* BHPKC28	8	16	23 ± 1

## Data Availability

The data presented in this study are available on request from the corresponding author.

## Supplementary Materials

The following supporting information can be downloaded at https://www.mdpi.com/article/10.3390/microorganisms13010180/s1, Figure S1—Dendrogram generated from the macrorestriction of genomes using XbaI after PFGE showing the % genetic similarity of *K. pneumoniae* isolates. The isolates in red were excluded from this study; Figure S2—Scheme of pBHKPC52_2 harboring *bla*_KPC_ gene in NTE_KPC_-IId. *bla*_KPC_ gene is shown in red. Other resistance genes are shown in orange. NTE_KPC_-IId genes are shown in yellow. Genes related to plasmid mobility capacity are shown in green. Replication genes are shown in blue; Figure S3—S1nuclease—PFGE gel of *K. pneumoniae* BHKPC10 (A), *E. coli* J53 (B), and transconjugant *E. coli* J53_pBHKPC10_2_3 (C). *Low-Range PFG Marker* (*New England Biolabs*, EUA) (M) was used as a molecular weight marker and was applied to both extremities of the gel. Table S1—Primers used for plasmid copy number determination; Table S2—Quality and coverage data from the genome sequencing of the 25 isolates and their annotated genome accession numbers; Table S3—Location of resistance genes and characterization of the plasmid populations present in each isolate of *K. pneumoniae*; Table S4—Location of resistance genes and characterization of the plasmid populations present in each isolate of *K. aerogenes*; Table S5—Location of resistance genes and characterization of the plasmid populations present in each isolate of *Enterobacter* spp.; Table S6—Location of resistance genes and characterization of the plasmid populations present in each isolate of *E. coli*; Table S7—Location of resistance genes and characterization of the plasmid populations present in each isolate of *P. stuartii*.
