# Supplementary material for: Dissemination of IncQ1 Plasmids Harboring NTEKPC-IId in a Brazilian Hospital
Source: Microorganisms. 2025 Jan 16;13(1):180. doi: 10.3390/microorganisms13010180 (PMC11767769; doi:10.3390/microorganisms13010180)
Supplement: Supplementary file 1 [file microorganisms-13-00180-s001.zip › Supplementary titles and figures.pdf]

Figure S1 - Dendrogram generated from the macrorestriction of genomes using XbaI after PFGE showing the % genetic similarity of *K. pneumoniae* isolates. The isolates in red were excluded from this study.

Figure S2 - Scheme of pBHKPC52_2 harboring *bla*KPC gene in NTEKPC-IId. *bla*KPC gene is indicated in red. Other resistance genes are shown in orange. NTEKPC-IId genes are shown in yellow. Genes related to plasmid mobility capacity are shown in green. Replication genes are shown in blue.

Figure S3 - S1nuclease - PFGE gel of *K. pneumoniae* BHKPC10 (A), *E. coli* J53 (B), and transconjugant *E. coli* J53_pBHKPC10_2_3 (C). *Low Range PFG Marker* (*New England Biolabs*, EUA) (M) was used as a molecular weight marker and was applied to both extremities of the gel.

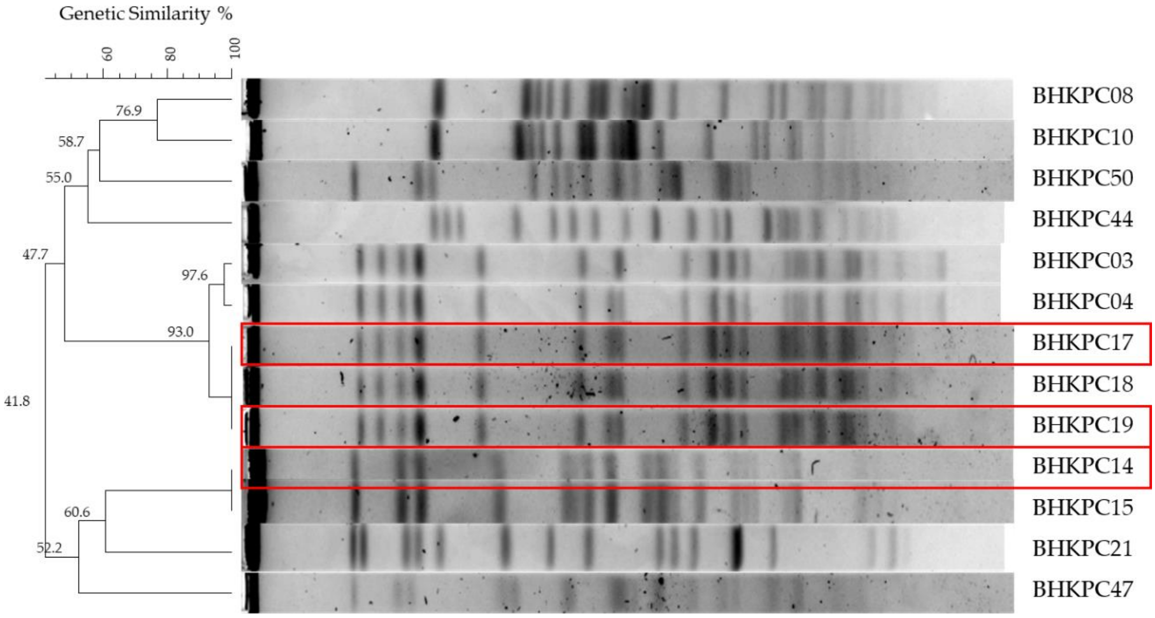

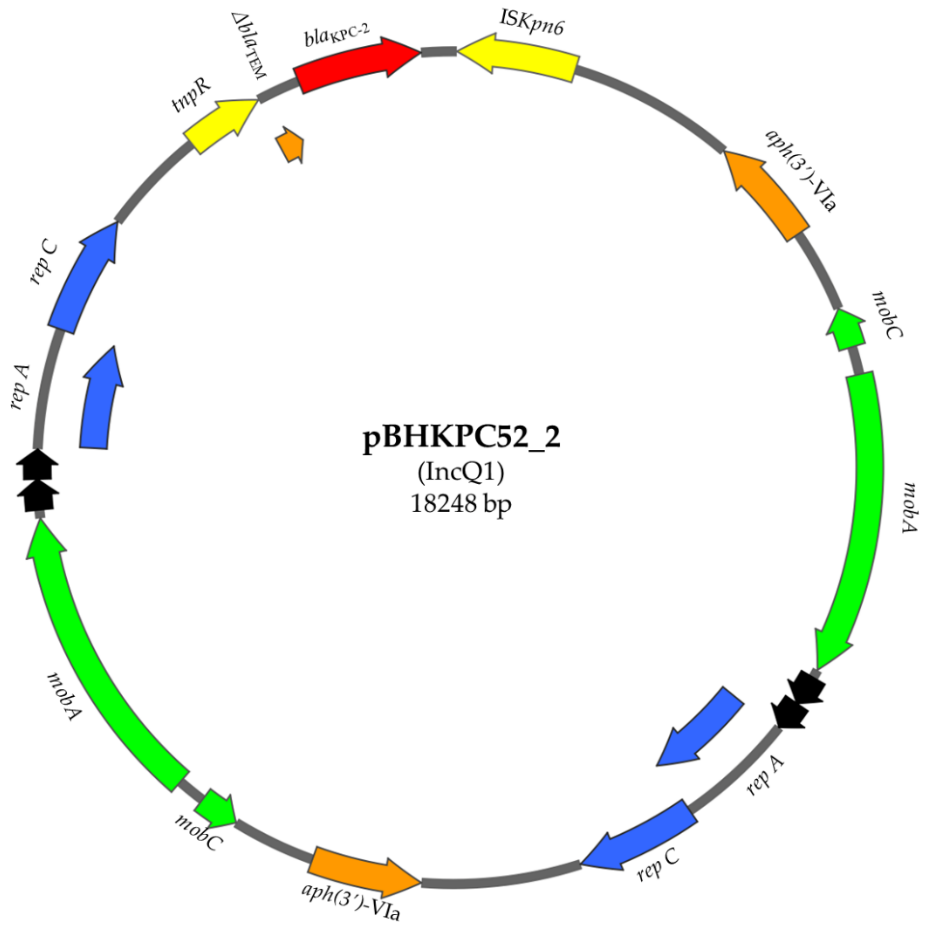
pBHKPC52_2
(IncQ1)
18248 bp
ISKpn6
bla KPC-2
Δbla TEM
tnpR
rep C
rep A
mobA
mobC
aph(3')-VIa
aph(3')-VIa
mobC
mobA
rep A
rep C

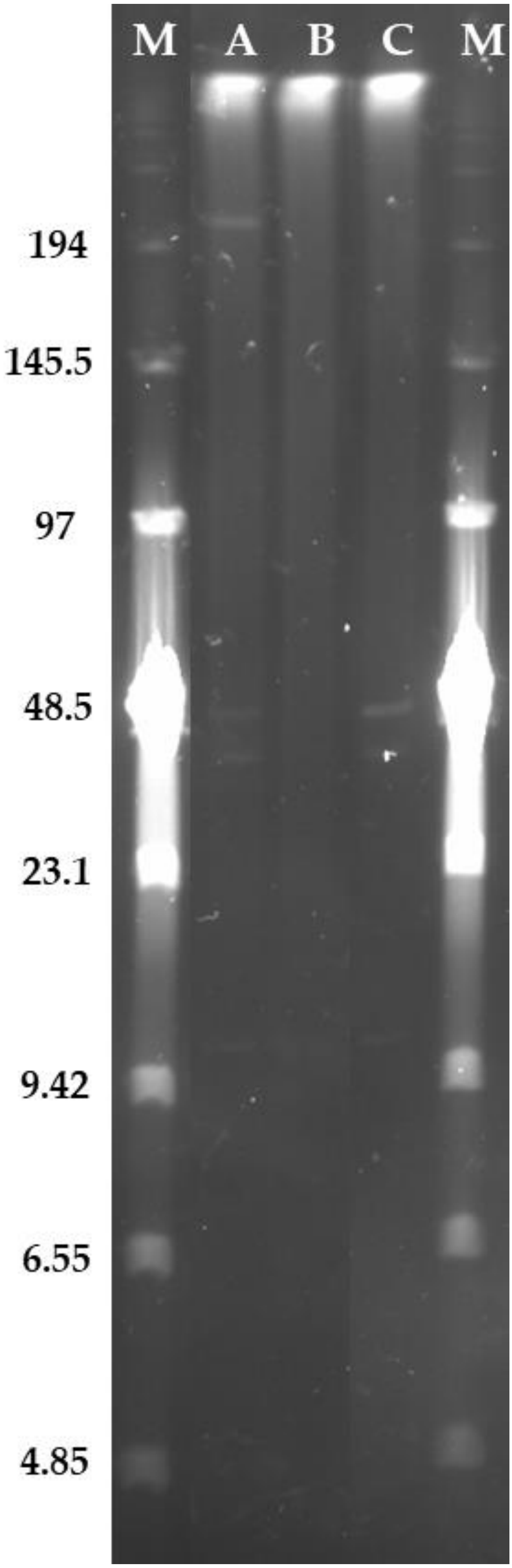
M A B C M
194
145.5
97
48.5
23.1
9.42
6.55
4.85
