# Supplementary material for: Dissemination of IncQ1 Plasmids Harboring NTEKPC-IId in a Brazilian Hospital
Source: Microorganisms. 2025 Jan 16;13(1):180. doi: 10.3390/microorganisms13010180 (PMC11767769; doi:10.3390/microorganisms13010180)
Supplement: Supplementary file 1 [file microorganisms-13-00180-s001.zip › TableS1.pdf]

Table S1 – Primers used for plasmid copy number determination.

Primer	Sequence (5′ – 3′)
*mdh_Ecoli*_F	CGATCTGAGCCATATCCCTACT
*mdh_Ecoli*_R	GAACGATCCATACCCGGTTTAC
*mdh_Kpneumoniae*_F	GCGGATGTAGTGCTGATCTC
*mdh_Kpneumoniae*_R	CTGCTGCACGAGGTTCTT
*mdh_Pstuartii*_F	CCTTAGCCATATCCCAACTGAC
*mdh_Pstuartii*_R	TACCTGGTTTACGAGCAACAC
*mdh_Kaerogenes*_F	GCATGGATCGTTCCGACTTA
*mdh_Kaerogenes*_R	CGATAGCGACGGTGGTATTT
*mdh_Enterobacter*_F	CTCAGAACTCTCCCTGTACGATA
*mdh_Enterobacter*_R	CACCGGAGAAGCCTTTGATT
bla_{KPC}_qPCR_F	GGCGGCTCCATCGGTGTGTA
bla_{KPC}_qPCR_R	AATTGGCGGCGGCGTTATCA
