# Supplementary material for: Dissemination of IncQ1 Plasmids Harboring NTEKPC-IId in a Brazilian Hospital
Source: Microorganisms. 2025 Jan 16;13(1):180. doi: 10.3390/microorganisms13010180 (PMC11767769; doi:10.3390/microorganisms13010180)
Supplement: Supplementary file 1 [file microorganisms-13-00180-s001.zip › TableS2.pdf]

Table S2 - Quality and coverage data from the genome sequencing of the 25 isolates and their annotated genome accession numbers.

Species	Isolate	Hospital	Coverage	N50	Accession numbers
Klebsiella pneumoniae	BHKPC03	HRTN	116x	197916	JAUPKA000000000
Klebsiella pneumoniae	BHKPC04	HRTN	43x	203715	JAUPKB000000000
Klebsiella aerogenes	BHKPC06	HRTN	145x	200304	JAUPKC000000000
Enterobacter hormaechei	BHKPC07	HRTN	32x	172684	JAUPMS000000000
Klebsiella pneumoniae	BHKPC08	HRTN	103x	208711	JAVCPB000000000
Klebsiella pneumoniae	BHKPC10	HRTN	260x	208711	JAVCPC000000000
Escherichia coli	BHKPC11	HRTN	116x	209759	CP132074-CP132075
Escherichia coli	BHKPC13	HRTN	178x	268668	CP132076-CP132077
Klebsiella pneumoniae	BHKPC15	HRTN	229x	373307	CP132078-CP132079
Klebsiella pneumoniae	BHKPC18	HRTN	152x	172898	JAVCPD000000000
Klebsiella pneumoniae	BHKPC21	HRTN	103x	241815	CP132080-CP132084
Providencia stuartii	BHKPC23	HRTN	153x	213454	CP132247-CP132248
Providencia stuartii	BHKPC27	HRTN	149x	78438	CP132249-CP132252
Enterobacter cloacae	BHKPC28	HRTN	84x	191661	JALKVG000000000
Providencia stuartii	BHKPC29	HRTN	124x	185696	CP132253-CP132254
Providencia stuartii	BHKPC30	HRTN	147x	284876	CP132255-CP132256
Providencia stuartii	BHKPC31	HRTN	183x	523668	CP132257-CP132258
Providencia stuartii	BHKPC35	HRTN	182x	157733	CP132259-CP132262
Escherichia coli	BHKPC37	HRTN	221x	200154	CP132085-CP132090
Providencia stuartii	BHKPC41	HRTN	105x	457351	CP132263-CP132264
Enterobacter hormaechei	BHKPC43	HRTN	47x	150982	CP132265-CP132270
Klebsiella pneumoniae	BHKPC44	HRTN	87x	323337	JAVCPE000000000
Klebsiella pneumoniae	BHKPC47	HRTN	167x	305448	JAVCPF000000000
Klebsiella pneumoniae	BHKPC50	HRTN	134x	322859	CP132091-CP132096
Klebsiella aerogenes	BHKPC52	HRTN	207x	261144	JAVCPG000000000
