# Supplementary material for: Dissemination of IncQ1 Plasmids Harboring NTEKPC-IId in a Brazilian Hospital
Source: Microorganisms. 2025 Jan 16;13(1):180. doi: 10.3390/microorganisms13010180 (PMC11767769; doi:10.3390/microorganisms13010180)
Supplement: Supplementary file 1 [file microorganisms-13-00180-s001.zip › TableS3.pdf]

Table S3 - Location of resistance genes and characterization of the plasmid populations present in each isolate of *K. pneumoniae*.

Chromosome/Plasmids	Contig	Classification	Resistance genes	Mobilization	Size (bp)
chromosome_BHKPC03	1	-	*fosA, OqxA,OqxB,*	-	5,437,498
pBHKPC03_1	2	IncFIB(pNDM-Mar) / IncHI1B(pNDM-MAR)	*aad1, aac(6')-Iq, sul2, sul1, dfrA15, sul1, aadA1, bla$_{SHV-182}$, bla$_{CTX-M-2}$, qacE*	Mobilizable	324,226
pBHKPC03_2	3	IncFII(K) / repB(R1701)	-	Conjugative	94,287
pBHKPC03_3	4	IncM1	-	Conjugative	73,682
pBHKPC03_4	5	IncR	-	Not mobilizable	22,698
pBHKPC03_5	8	IncQ1	*aph(3')-Via, bla$_{KPC-2}$*	Mobilizable	10,946
pBHKPC03_6	9	NI	-	Mobilizable	5,647
pBHKPC03_7	12	Col(pHAD28)	-	Mobilizable	4,096
chromosome_BHKPC04	1	-	*fosA, OqxB, OqxA, bla$_{SHV-182}$*	-	5,373,033
pBHKPC04_1	2	IncFIB(pNDM-Mar) / IncHI1B(pNDM-MAR)	*aadA1, aac(6')-Iq, sul1, dfrA15, sul2, bla$_{CTX-M-2}$, qacE*	Mobilizable	323,238
pBHKPC04_2	3	IncFII(K) / repB(R1701)	-	Conjugative	95,748
pBHKPC04_3	4	IncM1	-	Conjugative	73,628
pBHKPC04_4	6	IncR	-	Not mobilizable	49,739
pBHKPC04_5	8	IncQ1	*aph(3')-Via, bla$_{KPC-2}$*	Mobilizable	10,952
pBHKPC04_6	9	NI	-	Mobilizable	5,647
pBHKPC04_7	11	Col(pHAD28)	-	Mobilizable	4,096
chromosome_BHKPC08	1	-	*fosA, OqxA, OqxB, bla$_{SHV-33}$*	-	4,700,919
chromosome2_BHKPC08	2	-	-	-	533,736
pBHKPC08_1	3	IncFIB(K)	-	Conjugative	187,694
pBHKPC08_2	4	NI	-	Conjugative	54,440
pBHKPC08_3	5	IncQ1	*aph(3')-Via, bla$_{KPC-2}$*	Mobilizable	10,941
pBHKPC08_4	7	NI	-	Not mobilizable	3,679
chromosome_BHKPC10	1	-	*fosA, OqxB, OqxA, bla$_{SHV-33}$*	-	5,069,383
chromosome2_BHKPC10	3	-	-	-	158,775
pBHKPC10_1	2	IncFIB(K)	-	Conjugative	187,693
pBHKPC10_2	4	NI	-	Conjugative	54,440
pBHKPC10_3	5	IncQ1	*aph(3')-Via, bla$_{KPC-2}$*	Mobilizable	10,947
pBHKPC10_4	8	NI	-	Not mobilizable	3679
chromosome_BHKPC15	1	-	*fosA, OqxB, OqxA, bla$_{SHV-172}$*	-	5,321,796
pBHKPC15_1	2	IncQ1	*aph(3')-Via, bla$_{KPC-2}$*	Mobilizable	10,949

chromosome_BHKPC18	1	-	*fosA, OqxA, OqxB,* $bla_{SHV-182}$	-	5,455,033
pBHKPC18_1	2	IncFIB(pNDM-Mar) / IncHI1B(pNDM-MAR)	*aac(6')-Iq, aadA1, sul1, dfrA15, sul2,* $bla_{CTX-M-2}$, *qacE*	Mobilizable	326,748
pBHKPC18_2	3	IncFII(K) / repB(R1701)	-	Conjugative	90,171
pBHKPC18_3	4	IncM1	-	Conjugative	73,682
pBHKPC18_4	5	IncR	-	Not mobilizable	50,939
pBHKPC18_5	6	IncQ1	*aph(3')-Via,* bla_{KPC-2}	Mobilizable	10,950
pBHKPC18_6	7	NI	-	Mobilizable	5674
chromosome_BHKPC21	1	-	*fosA, OqxA, OqxB, blaSHV-40,* bla_{SHV-56}, bla_{SHV-89}, bla_{SHV-79}, bla_{SHV-85}	-	5,300,457
pBHKPC21_1	2	IncFIA(pBK30683) / IncFIB(K)(pCAV1099-114)	-	Conjugative	165,354
pBHKPC21_2	3	IncQ1	*aph(3')-Via,* bla_{KPC-2}	Mobilizable	10,946
pBHKPC21_3	4	Col440II	-	Mobilizable	5,252
pBHKPC21_4	5	NI	*qnrB19*	Mobilizable	3,947
chromosome_BHKPC44	1	-	*fosA, OqxB, OqxA,* $bla_{SHV-187}$	-	5,218,814
pBHKPC44_1	2	IncFIB(pKPHS1)	-	Not mobilizable	107,402
pBHKPC44_2	3	IncFIB(K) / IncFII(K)	-	Mobilizable	93,841
pBHKPC44_3	5	IncQ1	*aph(3')-Via,* bla_{KPC-2}	Mobilizable	10,948
pBHKPC44_4	6	Col440I	-	Not mobilizable	6,937
pBHKPC44_5	7	NI	-	Not mobilizable	4,773
-	4, 10, 11	-	*aph(6)-Id, aph(3'')-Ib, qnrB1, dfrA14, sul2,* $bla_{CTX-M-15}$, bla_{TEM-1B}, *aac(3)-Iia, aac(6')-Ib-cr,* bla_{OXA-1}, *catB3*	-	-
chromosome_BHKPC47	1	-	*OqxB, OqxA,* bla_{SHV-67}, bla_{SHV-11}	-	5,356,393
pBHKPC47_1	2	IncM2	*msr(E), fosA, mph(A), mph(E), qnrB4, sul1,* bla_{DHA-1}, *qacE*	Conjugative	82,937
pBHKPC47_2	4	IncR	*aph(3'')-Ib, aph(6)-Id,* $bla_{CTX-M-15}$	Not mobilizable	51,328
pBHKPC47_3	5	IncQ1	*aph(3')-Via,* bla_{KPC-2}	Mobilizable	10,952
pBHKPC47_4	7	NI	-	Mobilizable	3,223
pBHKPC47_5	8	Col(pHAD28)	-	Not mobilizable	2,495

chromosome_BHKPC50	1	-	*fosA*, *bla*$_{CTX-M-15}$, *bla*$_{SHV-121}$	-	5,386,786
pBHKPC50_1	2	IncFIB(K)	-	Conjugative	193,125
pBHKPC50_2	3	IncN/IncR	*aac(3)-Iia*, *aph(3'')-Ib*, *aac(6')-Ib-cr*, *aph(6)-Id*, *aadA5*, *mph(A)*, *dfrA17*, *sul1*, *aadA5*, *bla*$_{OXA-1}$, *bla*$_{CTX-M-15}$, *qacE*, *catB3*	Not mobilizable	62,588
pBHKPC50_3	4	NI	-	Not mobilizable	59,704
pBHKPC50_4	5	IncQ1	*aph(3')-Via*, *bla*$_{KPC-2}$	Mobilizable	10,949
pBHKPC50_5	6	NI	-	Not mobilizable	3,614

NI: not identified
