# Supplementary material for: Dissemination of IncQ1 Plasmids Harboring NTEKPC-IId in a Brazilian Hospital
Source: Microorganisms. 2025 Jan 16;13(1):180. doi: 10.3390/microorganisms13010180 (PMC11767769; doi:10.3390/microorganisms13010180)
Supplement: Supplementary file 1 [file microorganisms-13-00180-s001.zip › TableS4.pdf]

Table S4 - Location of resistance genes and characterization of the plasmid populations present in each isolate of *K. aerogenes.*

Chromosome/Plasmids	Contig	Classification	Resistance genes	Mobilization	Size (bp)
chromosome_BHKPC06	1	-	*fosA*	-	3,307,240
pBHKPC06_1	2	IncC	*aac(3)-Iia, qacE, sul1, dfrA22,* bla_{TEM-1B}*,* $bla_{CTX-M-2}$	Not mobilizable	83,448
pBHKPC06_2	3	IncM1	-	Conjugative	73,682
pBHKPC06_3	4	IncQ1	*aph(3')-Via,* bla_{KPC-2}	Mobilizable	10,948
pBHKPC06_4	5	ColRNAI	-	Mobilizable	9,294
chromosome_BHKPC52	1	-	*fosA*	-	5,436,500
pBHKPC52_1	2	IncC	*msr(E), aac(6')-Ib-cr, aac(3)-Iia, aac(6')-Ib3, mph(E), sul1,* $bla_{TEM-1B,}$ bla_{OXA-2}*, qacE*	Conjugative	153,890
pBHKPC52_2	3	IncQ1	*aph(3')-Via,* bla_{KPC-2}	Mobilizable	18,248
pBHKPC52_3	4	ColRNAI	-	Mobilizable	9,294
