# Supplementary material for: Dissemination of IncQ1 Plasmids Harboring NTEKPC-IId in a Brazilian Hospital
Source: Microorganisms. 2025 Jan 16;13(1):180. doi: 10.3390/microorganisms13010180 (PMC11767769; doi:10.3390/microorganisms13010180)
Supplement: Supplementary file 1 [file microorganisms-13-00180-s001.zip › TableS5.pdf]

Table S5 - Location of resistance genes and characterization of the plasmid populations present in each isolate of *Enterobacter* spp.

Chromosome/Plasmids	Contig	Classification	Resistance genes	Mobilization	Size (bp)
chromosome_BHKPC07	1	-	*fosA*, *bla*$_{ACT-7}$	-	4,803,210
pBHKPC07_1	2	NI	*aph(6)-Id*, *aadA1*, *aac(3)-Iia*, *aac(6')-Ib-cr*, *aph(3'')-Ib*, *qnrB1*, *dfrA14*, *sul2*, *tet(A)*, *bla*$_{CTX-M-15}$, *bla*$_{OXA-1}$, *bla*$_{TEM-1B}$, *catB3*, *catA1*	Mobilizable	234,132
pBHKPC07_2	3	IncQ1	*aph(3')-Via*, *bla*$_{KPC-2}$	Mobilizable	10,949
pBHKPC07_3	4	Col(pHAD28)	-	Mobilizable	4,096
chromosome_BHKPC43	1	-	*aadA1*, *fosA*, *dfrA1*, *sul1*, *bla*$_{ACT-16}$, *qacE*	-	4,890,604
pBHKPC43_1	2	IncHI2/IncHI2A	*aph(3'')-Ib*, *aadA1*, *aph(6)-Id*, *qnrB1*, *dfrA14*, *sul2*, *tet(A)*, *bla*$_{TEM-1B}$, *catA1*	Conjugative	309,583
pBHKPC43_2	3	IncFII(pCRY)	-	Conjugative	40,314
pBHKPC43_3	4	IncQ1	*aph(3')-Via*, *bla*$_{KPC-2}$	Mobilizable	10,948
pBHKPC43_4	5	NI	-	Mobilizable	3,223
pBHKPC43_5	6	Col(pHAD28)	-	Not mobilizable	2,495

NI: not identified
