# Supplementary material for: Dissemination of IncQ1 Plasmids Harboring NTEKPC-IId in a Brazilian Hospital
Source: Microorganisms. 2025 Jan 16;13(1):180. doi: 10.3390/microorganisms13010180 (PMC11767769; doi:10.3390/microorganisms13010180)
Supplement: Supplementary file 1 [file microorganisms-13-00180-s001.zip › TableS6.pdf]

Table S6 - Location of resistance genes and characterization of the plasmid populations present in each isolate of *E. coli*

**Chromosome/Plasmids**	**Contig**	**Classification**	**Resistance genes**	**Mobilization**	**Size (bp)**
chromosome_BHKPC11	1	-	*sitABCD*	-	4,817,895
pBHKPC11_1	2	IncQ1	*aph(3')-Via*, *bla*$_{KPC-2}$	Mobilizable	10,952
chromosome_BHKPC13	1	-	*aadA1*, *dfrA1*, *sul1*, *sitABCD*, *bla*$_{TEM-1A}$, *qacE*, *catA1*	-	5,275,482
pBHKPC13_1	2	IncQ1	*aph(3')-Via*, *bla*$_{KPC-2}$	Mobilizable	10,982
chromosome_BHKPC37	1	-	-	-	4,955,215
pBHKPC37_1	2	IncFIB(AP001918)	*aadA2b*, *aadA1*, *dfrA8*, *sul3*, *tet(A)*, *bla*$_{TEM-1B}$, *qacL*, *cmlA1*, *floR*	Conjugative	94,640
pBHKPC37_2	3	IncM1	-	Conjugative	68,006
pBHKPC37_3	4	IncQ1	*aph(3')-Via*, *bla*$_{KPC-2}$	Mobilizable	10,950
pBHKPC37_4	5	NI	-	Mobilizable	4,067
pBHKPC37_5	6	Col(pHAD28)	*qnrB19*	Not mobilizable	2,989

NI: not identified
