# Supplementary material for: Dissemination of IncQ1 Plasmids Harboring NTEKPC-IId in a Brazilian Hospital
Source: Microorganisms. 2025 Jan 16;13(1):180. doi: 10.3390/microorganisms13010180 (PMC11767769; doi:10.3390/microorganisms13010180)
Supplement: Supplementary file 1 [file microorganisms-13-00180-s001.zip › TableS7.pdf]

Table S7 - Location of resistance genes and characterization of the plasmid populations present in each isolate of *P. stuartii*

Chromosome/Plasmids	Contig	Classification	Resistance genes	Mobilization	Size (bp)
chromosome_BHKPC23	1	-	*aac(2')-Ia, catA3, tet(B)*	-	4,375,260
pBHKPC23_1	2	IncQ1	*aph(3')-Via, bla*$_{KPC-2}$	Mobilizable	10,949
chromosome_BHKPC27	1	-	*aac(2')-Ia, tet(B), catA3*	-	4,458,479
pBHKPC27_1	2	IncC	*aac(3)-Iia, aac(6')-Ib3, aac(6')-Ib-cr, sul1, bla*$_{TEM-1B}$, *bla*$_{OXA-2}$, *bla*$_{CTX-M-2}$, *qacE*	Conjugative	169,130
pBHKPC27_2	3	NI	-	Conjugative	36,059
pBHKPC27_3	4	IncQ1	*aph(3')-Via, bla*$_{KPC-2}$	Mobilizable	10,946
chromosome_BHKPC29	1	-	*aac(2')-Ia, tet(B), catA3*	-	4,376,968
pBHKPC29_1	2	IncQ1	*aph(3')-Via, bla*$_{KPC-2}$	Mobilizable	10,949
chromosome_BHKPC30	1	-	*aac(2')-Ia, tet(B), catA3*	-	4,374,916
pBHKPC30_1	2	IncQ1	*aph(3')-Via, bla*$_{KPC-2}$	Mobilizable	10,948
chromosome_BHKPC31	1	-	*aac(2')-Ia, tet(B), catA3*	-	4,374,698
pBHKPC31_1	2	IncQ1	*aph(3')-Via, bla*$_{KPC-2}$	Mobilizable	10,949
chromosome_BHKPC35	1	-	*aac(2')-Ia, tet(B), catA3*	-	4,458,749
pBHKPC35_1	2	IncC	*aac(3)-Iia,aac(6')-Ib3,aac(6')-Ib-cr,sul1,bla*$_{CTX-M-2}$,*bla*$_{TEM-1B}$,*bla*$_{OXA-2}$,*qacE*	Conjugative	169,130
pBHKPC35_2	3	NI	-	Conjugative	36,059
pBHKPC35_3	4	IncQ1	*aph(3')-Via,bla*$_{KPC-2}$	Mobilizable	10,949
chromosome_BHKPC41	1	-	*aac(2')-Ia, tet(B), catA3*	-	4,374,816
pBHKPC41_1	2	IncQ1	*aph(3')-Via, bla*$_{KPC-2}$	Mobilizable	10,949

NI: not identified
